# Prevalence Rates and Risk Factors for Primary Open Angle Glaucoma in the Middle East

**DOI:** 10.18502/jovr.v16i4.9755

**Published:** 2021-10-25

**Authors:** Rana Torabi, Alon Harris, Brent Siesky, Ryan Zukerman, Francesco Oddone, Sunu Mathew, Ingrida Januleviciene, Alice C. Verticchio Vercellin

**Affiliations:** ^1^Eugene and Marilyn Glick Eye Institute, Department of Ophthalmology, Indiana, University School of Medicine, Indianapolis, IN, USA; ^2^Icahn School of Medicine at Mount Sinai, New York, NY, USA; ^3^University of Miami Miller School of Medicine, Miami, FL, USA; ^4^IRCCS - Fondazione Bietti, Rome, Italy; ^5^Eye Clinic of Medical Academy of Lithuanian University of Health Sciences, Kaunas, Lithuania

**Keywords:** Epidemiology, Glaucoma, Middle East, Prevalence, Risk Factors

## Abstract

Glaucoma is a multifactorial disease and a leading cause of irreversible blindness worldwide. Current data has demonstrated the approximate distribution of primary open-angle glaucoma (POAG) in patients of European, African, Hispanic, and Eastern Asian descent. However, a significant gap in the literature exists regarding the prevalence of POAG in Middle Eastern (ME) populations. Current studies estimate ME POAG prevalence based on a European model. Herein we screened 65 total publications on ME prevalence of POAG and specific risk factors using keywords: “glaucoma”, “prevalence”, “incidence”, “risk factor”, “Middle East”, “Mideast”, “Persian”, “Far East”, as well as searching by individual ME countries through PubMed, Embase, Ovid, Scopus, and Trip searches with additional reference list searches from relevant articles published up to and including March 1, 2021. Fifty qualifying records were included after 15 studies identified with low statistical power, confounding co-morbid ophthalmic diseases, and funding bias were excluded. Studies of ME glaucoma risk factors that identify chromosomes, familial trend, age/gender, socioeconomic status, lifestyle, intraocular pressure, vascular influences, optic disc hemorrhage, cup-to-disc ratio, blood pressure, obstructive sleep apnea, and diabetes mellitus were included in this systematic review. We conclude that the prevalence of POAG in the ME is likely higher than the prevalence rate that European models suggest, with ME specific risk factors likely playing a role. However, these findings are severely limited by the paucity of population-level data in the ME. Well-designed, longitudinal population-based studies with rigorous inclusion and exclusion criteria are ultimately needed to accurately assess the epidemiology and specific mechanistic risk factors of glaucoma in ME populations.

##  INTRODUCTION

Glaucoma is a leading cause of irreversible blindness (defined as 6/120 visual acuity) and is responsible for up to 12% of all cases worldwide according to a 2014 meta-analysis by Tham et al.^[1,2]^ Glaucoma is a chronic, progressive optic neuropathy with a characteristic acquired optic nerve atrophy from retinal ganglion cell loss. There are two major subtypes of glaucoma: primary open-angle (POAG) and closed-angle glaucoma, both being based on the anterior chamber angle as demonstrated by gonioscopy.^[3]^ POAG is a multifactorial disease and a leading cause of irreversible blindness worldwide. Different populations present with different risk factors (RF) for and occurrence profiles for POAG. In recent years, glaucoma has been shown to disproportionately affect people of Asian and African descent (AD) compared to those of European descent (ED) (Table 1A).^[4]^ However, there have been few studies that focus specifically on the prevalence rates of glaucoma in the Middle Eastern (ME) population. In 2017, Alshawa et al identified the lack of extensive research into characterizing POAG in the ME.^[5]^ This is concerning given the extrapolation that by 2040 over 110 million people worldwide will be affected by some form of glaucoma, with a disproportionate impact on Asian and African patients.^[2]^ The prevalence of POAG and its RF have been well documented in patients from African, European, Hispanic, and Eastern Asian countries.^[1,2][6]^ This review seeks to determine the true prevalence rate of POAG in ME population. To the best of our knowledge, only two published studies specifically address ME patients in their POAG analysis – using a European model to create estimates for them.^[1,7]^

However, we postulate that POAG prevalence in ME patients is more similar to Asian countries given the geographical, lifestyle, and epidemiological similarities they share. POAG prevalence rates in Asia are considerably higher than European model estimates (2.82%; 95% confidence interval, CI, 1.67-3.06% versus 1.47%; 95%CI 1.06-2.06%; Table 1A), and reported clinical data from ME countries shows a higher prevalence of POAG than European estimates (Table 1B).

Due to the multi-factorial nature of POAG, a more specific understanding of ME geographic-specific RF is also important for improving long-term POAG management. RF for POAG have been studied at length; however, there is a gap in the literature with regards to the RF of glaucoma in ME patients. These RF may possibly include demographics such as age, gender, lifestyle, or genetics; as well as other ocular parameters such as intraocular pressure (IOP), vascular influences, optic disc hemorrhage (DH), cup-to-disc ratio (C/D); and systemic comorbidities such as blood pressure (BP) and anti-hypertensive medications, obstructive sleep apnea (OSA), and diabetes mellitus (DM).

Overall, the goal of this review is to discuss current knowledge of POAG in ME countries, and to explore RF that may increase risk, or conversely be protective, for the development and progression of glaucoma in ME populations.

##  METHODS

PubMed, Embase, Ovid, Scopus, and Trip searches were conducted from all available published articles through March 1, 2021 using MeSH searches involving the terms “glaucoma”, “prevalence”, “incidence”, “risk factor”, “age”, “gender”, “lifestyle”, “genetics”, “IOP”, “DH”, “C/D”, “BP”, “OSA”, “DM”, “Middle East”, “Mideast”, “Persian”, “Far East”, as well as searching by individual ME countries (i.e., Saudi Arabia, Bahrain, Kuwait, Iraq, Syria, Oman, United Arab Emirates, Jordan, Qatar, Lebanon, Egypt, Iran, Yemen, Palestine, and Israel) to evaluate all pertinent articles, abstracts, and ongoing research projects. Example of MeSH search from PubMed: { (Glaucoma [MeSH Terms]) AND (Prevalence [MeSH Terms]) } AND (Iran [MeSH Terms]).

**Figure 1:**
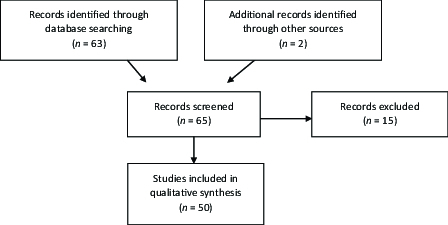
Flow chart showing method of literature search and results of literature review.

Reference lists from all relevant articles were reviewed to crossmatch and capture all pertinent articles. Sixty-five records were screened and fifty were included in the qualitative synthesis [Figure 1]. All studies were published in English. Gray literature was not included. Due to the paucity of available articles, a wide variety of studies were included (i.e., cross-sectional, population based, review, prospective cohort, and random quota sampling.) In order to maximize the quality of this review, only journal articles with clinically significant differences in chromosomal factors, familial trends, ocular factors, and systemic comorbidities of interest were included in this systematic review by various members of our team. Articles that did not meet those standards or had low sample size (*n* < 500), confounding variables (e.g., co-morbid ophthalmic diseases), or funding bias were excluded.

**Table 1:** Current worldwide estimates of primary open-angle glaucoma including: (A) Middle Eastern (ME) prevalence which is based on the European model. (B) Revised ME and worldwide prevalence which is based on the clinical studies presented in this study^[3]^


**World region**	**Ratio glaucoma to population > 40**	
	**(A) ME prevalence based on European model**	**(B) ME prevalence based on published clinical data in the literature**
**Middle East**	1.47%	3.07%
**Europe**	2.23%	
**Southeast Asia**	2.38%	
**India**	2.55%	
**China**	2.66%	
**Latin America**	3.35%	
**Japan**	3.70%	
**Africa**	4.32%	
**World**	**2.65%**	**3.03%**
	
	

**Table 2:** Summary of articles relating to primary open-angle glaucoma prevalence in Middle Eastern countries.


**Study**	**Authors**	**Year of publication**	**Sample size**	**Age of patients**	**Prevalence of POAG**
*Prevalence of glaucoma types and legal blindness from glaucoma in the western region of Saudi Arabia: a hospital-based study *	Eid TM, El-hawary I, El-menawy W.^[7]^	2009	2,354	≥ 20 years	30.5%
*Prevalence and Determinants of Glaucoma in Citizens of Qatar Aged 40 Years or Older: A Community-Based Survey *	Al-Mansouri FA, Kanaan A, Gamra H, et al.^[8]^	2011	3,149	≥ 40 years	1.71%
*Oman Eye Study 2005: prevalence and determinants of glaucoma *	Khandekar R, Jaffer MA, Al Raisi A, et al.^[9]^	2008	3,324	≥ 30 years	4.75%
*The prevalence and causes of blindness in the Sultanate of Oman: the Oman Eye Study (OES) *	Khandekar R, Mohammed AJ, Negrel AD, et al.^[10]^	2002	11,417	0 to +60 years	1.02%
*Glaucoma in oman: a review *	Khandekar R, Zutshi R.^[11]^	2006	Estimate based on population of 1,869,580	≥ 40 years	1.14/1000
*The Maccabi Glaucoma Study: Treatment Patterns and Persistence with glaucoma therapy in a large health organization in Israel *	Goldshtein I, Levkovitch-Verbin H, Shalev V, et al.^[12]^ And Levkovitch-Verbin H, Goldshtein I, Chodick G, et al.^[13]^	2013	5,934	≥ 45 years	1.61%
*A Population-based Survey of the Prevalence and Types of Glaucoma in Central Iran: The Yazd Eye Study *	Pakravan M, Yazdani S, Javadi M-A, et al.^[14]^	2013	2,098	40-80 years	4.4%
*Prevalence and risk factors of glaucoma in an adult population from Shahroud, Iran *	Hashemi H, Mohammadi M, Zandvakil N, et al.^[15]^	2018	6,311	40-67 years	1.92%
*Prevalence and causes of visual impairment among Saudi adults attending primary health care centers in northern Saudi Arabia *	Al-Shaaln FF, Bakrman MA, Ibrahim AM, et al.^[17]^	2011	617	≥ 18 years	5.8%
*The prevalence and determinants of glaucoma among 40 Years and Older Saudi Residents in the Riyadh Governorate (except the Capital) – A community based survey *	Khandekar R, Chauhan D, Yasir ZH, et al.^[18]^	2019	940	≥ 40 years	5.6%
	
	

**Table 3:** Prevalence of the different types of glaucoma in the Middle East^[5,7,8,9,10,11,12,13,14,15,16,18]^


	**Iran**		**Israel**		**Oman**	**Saudi Arabia**	**Qatar**
**POAG**	**NTG**	3.20%	1.70%	1.61%	1.55%	1.93%	2.92%	0.78%
	**HTG**	1.50%	0.06%		
**Angle closure suspect**		0.11%		0.11%		1.80%	2.71%	0.16%
**Other glaucoma**		0.40%		0.17%		1.02%	0.17%	0.27%
**Total Glaucoma**		4.40%		2.20%		4.75%	5.80%	1.71%
HTG, high tension glaucoma; NTG, normal tension glaucoma; POAG, primary open angle glaucoma				

**Table 4:** Average Cup-to-Disc ratios in patients of African, European, and Asian descent^[46,47,48]^


	**Cup-to-Disc ratio (C/D)**
**African descent**	**0.35**
**European descent **	**0.24**
**Korea**	**0.34 +/– 0.12**
**China**	**0.44 +/– 0.17**
**Singapore**	**0.40 +/– 0.15**

**Table 5:** Summary of possible risk factors of primary open angle glaucoma in Middle Eastern countries versus non-Middle Eastern countries


**Risk factors**	**Non-Middle Eastern countries**	**Middle Eastern countries**
**Chromosomes **	**O**	**O^[32,33,34,35,36]^**
**Familial trends**	**O**	**?**
**Age and gender**	**O**	**O^[14,17]^**
**Socioeconomic status**	**O**	**O^[14,17]^**
**Lifestyle**	**O**	**?**
**Intraocular pressure**	**O**	**O^[44]^**
**Vascular **	**O**	**?**
**Optic disc hemorrhage**	**O**	**?**
**Cup-to-Disc ratio**	**O**	**?**
**Blood pressure**	**O**	**?**
**Obstructive sleep apnea**	**O**	**?**
**Diabetes mellitus**	**X**	**X^[59]^**
Key: X, no association; O, association; ?, unclear/unknown association		

**Figure 2:**
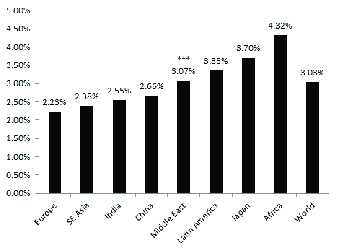
Graphical representation of worldwide primary open-angle glaucoma and angle closure glaucoma prevalence rates.

##  RESULTS

### Epidemiology

The average prevalence rate of POAG in ME countries was based off clinical studies conducted in the region of interest. We specifically included Iran, Israel, Qatar, Oman, and Saudi Arabia (SA) in our calculations due to data availability. A summary of the major ME articles relating to POAG prevalence have also been listed [Table 2]. Average prevalence of high-tension glaucoma (HTG), normal-tension glaucoma (NTG), acute angle closure glaucoma (ACG), and other forms were calculated by dividing the number of affected individuals by the number of individuals in the studied population at that particular time [Table 3].^[6,8,9,10,11,12,13,14,15,16,17,19]^

While worldwide rates of blindness secondary to glaucoma are predicted to decrease from 8.49 (95% CI 2.99–15.66) in 2015 to 8.43 (95% CI 2.75–15.96) in 2020, the prevalence in the ME are expected to keep rising from 6.89 (95% CI 2.20–13.16) to 6.94 (95% CI 2.04–13.57).^[20]^

We compared five ME countries to approximate the prevalence of glaucoma and we estimate true ME POAG rates to be lower than patients of African or Latino descent but greater than Caucasian and East Asian (EA) patients [Figure 2].

Immigrants from the ME make up a considerable amount of the United States immigrant population with a 36% increase witnessed from 2010–2016 to over 1.1 million lawful permanent residents.^[21]^ Given the geographic diversity of the ME, some of the RF for developing POAG specific in subset regions like North Africa or West Asia (structural ocular differences, IOP, myopia) could also be applied to patients of ME descent.^[4,22,23]^

### Genetic Characteristics

#### Chromosomal influences 

In 2014, Takamoto et al identified several genome-wide candidate genes that may be involved, including the S1 RNA binding-domain region on chromosome 2p21 and the caveolin regions on 7q31, to name a few.^[24]^ Similarly, in 2015, Nakano et al identified three genetic loci through a genome-wide association study (GWAS) associated with POAG in a Japanese population.^[25]^ Follow-up of these alleles, and several others, in an ME population, however, has led to largely negative associations with POAG.^[26,27,28,29,30,31]^

More recently, Kondkar et al identified several polymorphisms of the endothelial nitric oxide synthase (*NOS3*) gene that may modify the risk of POAG in the SA population, possibly supporting the oxidative stress hypothesis for the pathogenesis of POAG.^[32]^ Additionally, polymorphism of the *SIX1*/*SIX6* gene locus has been identified in SA (genotype distribution, *p* = 0.012).^[33]^ Meanwhile in Iran, a cohort study of 65 unrelated patients had a statistically significant increased occurrence of POAG and the p53 Pro72 allele (*p* < 0.05),^[34]^ and a more recent Iranian study demonstrated that polymorphisms of the IL-10 gene promoter are significantly associated with susceptibility to POAG similar to findings demonstrating IL-10 polymorphisms to be predictive of POAG pathogenesis in China.^[35,36]^ Finally, a 2019 GWAS connecting the *APBB2* gene with POAG risk in patients with African ancestry included individuals from SA (as individuals with African ancestry admixture), indicating that the *APBB2* gene polymorphisms associated with POAG may have further association with the greater ME population.^[37]^

#### Familial trends

Glaucoma is associated with familial trends. The Baltimore Eye Study conducted a population-based survey and demonstrated higher rates of glaucoma in siblings (odds ratio, OR = 3.69) than parents (OR = 2.17) or children (OR = 1.12).^[38]^ No recent publications from the ME have evaluated familial trends of glaucoma except for a single cross-sectional observational study of ACG in Iranian siblings which showed increased risk of ACG among siblings (57.9%; 95% CI 52.6–73.7).^[39]^

### Demographic Risk Factors

#### Age and gender

In 2013, a review of glaucoma rates in central Iran showed a ratio of 37:50 between men and women in POAG occurrence.^[14]^ This matches a survey of the United States that showed women to have higher self-reported visual impairment from glaucoma than men (OR = 1.20; 95% CI 0.99–1.45) and, generally, worldwide data on glaucoma incidence.^[40,41]^

#### Socioeconomic status

Prevalence of POAG in central Iran are stratified by socioeconomic status and education, with 4% prevalence in urban and 7.7% prevalence in rural communities and more than 80% of the glaucoma population at < 6 ${}^{\mathrm{th}}$ grade education.^[14]^ A similar study in SA demonstrated 48.9% of the POAG population to be illiterate, and 42.3% unemployed.^[17]^ This overall trend matches a 2015 UK study which correlated individuals reporting glaucoma with more adverse socioeconomic characteristics, based on the Townsend deprivation index.^[42]^

#### Lifestyle

In 2013, a multivariate model distinguished certain lifestyle characteristics that may be associated with higher rates of POAG in the general population. Certain factors, such as alcohol usage (not associated with POAG; *p* < 0.05), matched existing literature.^[43]^ Interestingly, heavy tobacco usage ( ≥ 40 pack-years) showed an association with increased POAG rates (OR 3.93; 95% CI 1.12–13.80; *p* = 0.03).^[43]^ Currently no studies have investigated tobacco usage in ME POAG patients.

### Ocular Risk Factors

#### Intraocular pressure (IOP)

Many patients with glaucomatous visual impairment experience disease progression despite physiologic or medically lowered IOP. Specifically, Asian epidemiologic studies have evaluated 52–92% of POAG cases to have NTG.^[23]^ The rates of NTG, however, varies widely among different patient population groups (57.1% in AD vs 20–28.9% in ED) despite similar rates of POAG (2.02+/–1.21%). Interestingly, even mean IOP varies among Asian countries; mean IOP values assessed in Japan were 13.1 mmHg (standard deviation, SD = 3.0) and 16.1 mmHg in Korea (SD = 3.2).^[23]^

In the ME, a comprehensive study of IOP in healthy (non-glaucomatous) Iranian patients found a mean IOP of 14.5 mmHg (SD = 2.5).^[44]^ No studies comparing NTG or HTG were found.

#### Vascular influences

Reductions in the retinal capillary and retrobulbar blood flow (BF) have been shown to strongly correlate with changes in optic nerve head (ONH) and macular thickness in POAG patients of AD.^[4]^ This data suggests that ocular vascular health may be a pathophysiologic explanation for the higher rates of POAG seen in patients of AD versus ED.^[4]^ Studies of BF and bulk perfusion have not been published in ME population.

#### Optic disc hemorrhages (DH)

DH flame-shaped bleeds typically occur around the temporal portion of the optic disc. In 2003, Gazzard et al analyzed a cohort of 167 South East Asian patients with POAG and found only 5 cases of DH, with 80% associated with POAG.^[45]^ Overall, they reported a level of DH prevalence as 2.99%, which is comparable to rates in Caucasian patients at 2.44%. Interestingly, DH were not seen as frequently in NTG patients in their study.^[45]^ Whether or not these values can be replicated in a larger study and also be applied to ME populations has yet to be determined.

#### Cup-to-disc ratio (C/D)

Increased C/D is a known RF for the progression of glaucoma (0.3 healthy, 0.7 glaucomatous). As early as 1985, Beck et al recorded an average C/D of 0.35 in AD patients and 0.24 in Caucasian patients (*p* < 0.0001) showcasing higher vertical C/D ratios for AD patients.^[46]^ Currently such studies have not been performed in ME patients.

### Systemic Comorbidities

#### Blood pressure (BP) and anti-hypertensive medications

While BP control is an essential part of care for many patients, hypertensive medications may be associated with glaucoma progression.^[47]^ In 2017, a study of patients from several ME countries reported a prevalence of HT at 31%.^[48]^ At the time, 47% of this group was receiving antihypertensive treatment – with only 19% successfully reaching physiologic BP. Specifically, older women had a higher frequency of systemic arterial hypertension (HT) treatment than men and younger patients (*p* < 0.0001) suggesting a correlation between prevalence of glaucoma and overtreatment of HT in female patients.^[48]^

The link between lowered BP and glaucoma progression is supported by the Thessaloniki Eye Study, which used tomography to measure the optic disc while recording systolic BP (SBP) and diastolic BP (DBP). Stratification for BP and antihypertensive drug classes showed that DBP < 90 mmHg led to increased C/D.^[49]^

Similarly, a meta-analysis from the USA suggests physiological dips in nocturnal BP may lead to glaucoma progression over a two-year span (OR 3.32 [1.84–6.00] DBP; OR 2.09 [1.20–3.64] SBP).^[50]^

#### Obstructive sleep apnea (OSA)

A 2019 study by Fan et al demonstrated a significantly increased risk of RNFL thinning progression (hazard ratio, HR, 8.448, 95% CI 1.464–48.752, *p* = 0.017) in Taiwanese patients with severe OSA, indicative of a higher risk of glaucomatous structural deterioration.^[51]^ Recent prevalence studies and meta-analyses have also demonstrated a correlation between severe OSA and glaucoma; in 2015, Wu et al demonstrated a significantly increased risk of glaucoma in patients with OSA/hypopnea syndrome (OSAHS) (OR = 1.65, 95% CI 1.44–1.88; *I ${}^{2}$* = 43%).^[52,53,54]^

As for POAG specifically, in 2018 Friedlander et al demonstrated a significant association between POAG and OSA, noting a prevalence rate of 20.49% in patients with OSA when compared to the general population (2.5%) among a study sample of 225 patients (*p* < 0.00001).^[55]^ Additionally, a subgroup analysis of a 2015 meta-analysis from Wu et al demonstrated increased POAG risk among OSA patients (OR = 1.87, 95% CI 1.54–2.33, *I ${}^{2}$* = 0%).^[52]^ There is only one 2019 study from SA, which demonstrates the prevalence of glaucoma in SA OSA patients was higher than in similar studies conducted in Europe and Asia, but less than the United States, suggesting a possible geographic contribution.^[56]^

#### Diabetes mellitus (DM)

Over 343 million people worldwide are affected by type II DM, and the ME has the second highest rate of increase in DM.^[57]^ POAG and DM are both diseases with vascular components, and DM may portend to the progression of POAG by predisposing patients to vascular autoregulatory dysfunction and impaired release of endothelial factors in retinal, choroidal, and retrobulbar circulation.^[58]^

In the ME, however, a small study in Yemen evaluating glaucomatous visual impairment in diabetic patients found only 4.4% of patients to have concurrent glaucoma and DM; other ocular diseases occurred more frequently in the DM ME population.^[59]^ Similarly, a European GWAS demonstrated no correlation between DM- and glaucoma-related traits.^[60]^

##  DISCUSSION

Glaucoma is a leading cause of irreversible blindness that with careful monitoring can be managed. However, current studies in the literature have not specifically studied the ME population. Based on current data we suggest the ME has a higher POAG prevalence than previously documented in the literature, which is based on European model instead of rigorous, clinical studies. We also explore factors that may increase risk, or conversely be protective, for the development and progression of glaucoma in ME populations. Table 5 summarizes current knowledge about known RF in ME versus non-ME countries. Based on the aforementioned studies, due to lack of published studies the RF of familial trends, lifestyle, ODH, vascular, C/D, BP, and OSA are termed as having an unclear association between POAG and the ME population.

### Genetic Characteristics

Many genes have been implicated in the development of POAG and given the rate of consanguinity/endogamy in the ME, warrant significant consideration in this population. As previously mentioned by Nakano et al, several GWAS of the Japanese population have identified specific alleles with a strong association with glaucoma.^[24]^ At this time, however, there are only isolated studies looking at specific chromosomal factors and their relationship to glaucoma prevalence or its pathogenesis. Current studies performed on ME patients do not readily point to an all-encompassing genetic correlation of POAG rates between ME, European, or Asian patients. Such studies are important and could be the next step in developing stricter screening guidelines for at-risk families, especially those who are not aware of their medical family history.

There are certain demographic characteristics that may also lead to higher rates of glaucoma in the Middle East. POAG is already commonly known to be associated with an aging population, but features like gender, lifestyle, and socioeconomic status may also play a role in prevalence and progression rates. There are few studies reporting age/gender RF for ME patients. However, preliminary data seems to suggest a gender bias for aging women to have higher rates of reported visual impairment.^[12]^ This may simply be due to reporting bias, gender inequality for accessing healthcare, or possible loss of protective factors such as estrogen as women age. Another factor is socioeconomic status. Individuals with less education or those in a more rural home environment portend to have a higher prevalence of POAG.^[12,15,41]^ The data suggests a possible correlation to a lack of adequate preventive healthcare, leading to more diagnoses of POAG. Lastly, lifestyle characteristics may be a significant RF. Alcohol was not associated with increased prevalence of POAG; however, according to our literature search tobacco has been studied and is correlated with higher POAG prevalence.^[42]^ This is particularly concerning for the ME, as Arab nations are experiencing a tobacco epidemic with approximately half of all men reporting daily smoking habits, and half of this population smoking > 20 cigarettes/day.^[61]^

### Ocular Risk Factors

There are certain physiological parameters of the eye itself that predispose to the occurrence of glaucoma including, but not limited to, IOP, DH, and C/D ratios.

IOP remains the only approved modifiable treatment target in POAG. It is well-known that Asian populations have a higher incidence of NTG.^[22]^ Since we are estimating that ME patients have a closer glaucoma prevalence to Asian population, it would be interesting to study whether or not there is a higher prevalence of HTG or NTG in the ME population. Per our literature search, this type of study has not been conducted yet.

Decreased capillary BF may also correlate with POAG. Vascular health, however, is complicated and multifactorial. Bulk perfusion, or ocular perfusion pressure (OPP), is related to mean arterial pressure (MAP) and IOP through the formula OPP = MAP – IOP. As such, vascular insult can occur during IOP and BP fluctuations, both of which are mediated by vascular autoregulation and can affect glaucoma prevalence.^[3]^ Ultimately, this interplay between OPP and BP may explain the pathophysiology of POAG patients even with medication controlled IOP, especially those found in EA and ME countries.^[62,63]^ In regard to DH, these may be an RF for glaucoma progression due to structural and functional damage from the bleed, specifically POAG versus NTG.

Lastly, postulations have been made between glaucoma and its connection with increased disc cupping. Similar results of increased C/D can be seen when comparing the prevalence of C/D between various Asian nations with comparable vertical cupping of approximately 0.39 in Korean, Chinese, and Singaporean populations [Table 4].^[64,65,66]^ Using a multiple regression analysis, positive associations for increased C/D (*p* < 0.001) were observed with factors such as increasing age, increasing IOP, and decreased diastolic BP, while body mass index had a negative association.^[65,66]^ Overall, the prevalence of increased disc cupping in Asians was comparable to patients of AD despite their lower rates of glaucoma.^[65,66]^ Despite its high association with glaucoma progression in AD patients, the same increase of C/D was not noted to have a similar rise in POAG in Asian patients when normalizing for other confounding variables. Such correlations question whether or not C/D is a significant determinant of glaucoma risk in patients of Asian or ME descent.

### Systemic Comorbidities

Certain systemic illnesses have also been postulated to affect the incidence and progression of glaucoma including systemic arterial HT, OSA, and DM.

HT and aggressive BP control can lead to glaucoma progression as shown in the Thessaloniki Eye Study and the Bowe et al group.^[54,55]^ This data warrants evaluation in Asian and ME patients with physiologic IOP as it suggests low BP as a pathogenetic factor for glaucoma.

In regard to OSA in the ME, data is generally lacking about OSA, but available data suggests a dramatic increase in the prevalence of OSA, likely due to an uncontrolled obesity epidemic. Epidemiologically, it is estimated that 33.3% of SA patients, 16.8% of Jordanian patients, 27.3% of Iranian patients, and 20.9% of patients in Dubai should be considered high-risk for OSA.^[67,68,69,70,71]^ Still, there is limited data describing the interplay of OSA and glaucoma in the ME.

There is currently a high rate of DM in the ME, which has been shown to influence and likely dysregulate vascular endothelial factors affecting BF. Previous data showed that changes in retinal capillary BF correlated more strongly with changes in ONH morphology in patients with DM, suggesting that changes in retinal BF may play a larger role in glaucomatous disease progression in patients with POAG with diabetes. However, current studies reviewing the prevalence of POAG have not shown a significant association between glaucoma and DM, suggesting that the high prevalence of DM in the ME may not be a large RF for glaucoma.^[19,72,73,74]^ Given the limited nature of ME studies, however, it is possible for either hypothesis to be true.

##  Limitations

In reviewing the prevalence of glaucoma and potential role of RF in the ME population, there are significant challenges and limitations to acknowledge. First, there is the relative ambiguity of the definition of the ME, which generally includes West Asia and North Africa, but sometimes includes Turkey, the South Caucuses, Afghanistan, and Pakistan.^[75]^ Notably, prevalence data was not available from all ME countries – including Jordan and Kuwait (though glaucoma has become the leading cause of registered blindness in the Kuwaiti population over 60).^[76]^ For similar reasons, North Africa was excluded from review. Importantly, the definition of glaucoma also varies among studies – generally following World Health Organization (WHO) definitions or using more arbitrary IOP values to define disease. Similarly, population sizes and characteristics (e.g., age range) varied between studies. Given the paucity of robust, uniform randomized-controlled clinical studies in ME populations, a limitation of this review includes not being able to present data as a meta-analysis. Lastly, selection bias may also affect studies with data collected from local/state hospitals and may be less representative of an entire country's population.

##  SUMMARY

Despite growing prevalence of glaucoma globally, studies investigating glaucoma in ME populations are underrepresented. Of the available data, many studies have methodological limitations affecting the consensus of published studies. European modeling to estimate ME prevalence rates likely underestimate true values. Accurate POAG rates in ME populations are likely higher than in ED and EA countries, and lower than AD or LAD populations. Similarly, RF presented in this review may influence ME rates of POAG, but data is lacking to confirm their role in disease pathogenesis. Early associations have been found between the factors of chromosomes, age/gender, socioeconomic status, IOP, and vascular influences in ME population. No known associations have been found linking DM. RF including familial trends, lifestyle, OD hemorrhages, CDR, BP, and OSA have unclear associations such that an association can be proved or disproved at this time for ME population.

Therefore, we encourage well-designed, longitudinal population-based ME studies with carefully considered inclusion and exclusion criteria to accurately assess the epidemiology of glaucoma and specific mechanistic RF in ME populations for better outcomes and improvement in quality of life.

##  Financial Support and Sponsorship

Alon Harris was supported by NIH grant (R01EY030851), NSF DMS (1853222/2021192), and in part by a Challenge Grant award from Research to Prevent Blindness, NY.

##  Conflicts of Interest

The authors declare no competing interests. Dr. Alon Harris would like to disclose that he received remuneration from AdOM, Qlaris, Luseed, and Cipla for serving as a consultant, and he serves on the board of AdOM, Qlaris, and Phileas Pharma. Alon Harris holds an ownership interest in AdOM, Luseed, Oxymap, Qlaris, Phileas Pharma, and QuLent. All relationships listed above are pursuant to Icahn School of Medicine's policy on outside activities. The contribution of the author Dr. Francesco Oddone was supported by Fondazione Roma and by the Italian Ministry of Health. None of the other authors listed have any financial disclosures.

## References

[B1] Quigley HA, Broman AT (2006). The number of people with glaucoma worldwide in 2010 and 2020. Br J Ophthalmol.

[B2] Tham Y-C, Li X, Wong TY, Quigley HA, Aung T, Cheng C-Y (2014). Global prevalence of glaucoma and projections of glaucoma burden through 2040: a systematic review and meta-analysis. Ophthalmology.

[B3] Prum BE, Rosenberg LF, Gedde SJ, Mansberger SL, Stein JD, Moroi SE, et al (2016). Primary open-angle glaucoma preferred practice pattern guidelines. Ophthalmology.

[B4] Huck A, Harris A, Siesky B, Kim N, Muchnik M, Kanakamedala P, et al

[B5] Alshawa L, Harris A, Gross J, Snyder A, Rao A, Siesky B (2017). Primary open-angle glaucoma in patients of Middle Eastern descent. Saudi J Ophthalmol.

[B6] Cheng J-W, Zong Y, Zeng Y-Y, Wei R-L (2014). The Prevalence of primary angle closure glaucoma in adult Asians: a systematic review and meta-analysis. PLoS One.

[B7] Eid TM, El-hawary I, El-menawy W (2009). Prevalence of glaucoma types and legal blindness from glaucoma in the western region of Saudi Arabia: a hospital-based study. Int Ophthalmol.

[B8] Al-Mansouri FA, Kanaan A, Gamra H, Khandekar R, Hashim SP, Al Qahtani O, et al (2011). Prevalence and determinants of glaucoma in citizens of Qatar aged 40 years or older: a community-based survey. Middle East Afr J Ophthalmol.

[B9] Khandekar R, Jaffer MA, Al Raisi A, Zutshi R, Mahabaleshwar M, Shah R, et al (2008). Oman Eye Study 2005: prevalence and determinants of glaucoma. East Mediterr Health J.

[B10] Khandekar R, Mohammed AJ, Negrel AD, Al Riyami A (2002). The prevalence and causes of blindness in the Sultanate of Oman: the Oman Eye Study (OES). Br J Ophthalmol.

[B11] Khandekarl R, Zutshi R (2006). Glaucoma in Oman: a review. J Glaucoma.

[B12] Goldshtein I, Levkovitch-Verbin H, Shalev V, Zigman N, Chodick G (2013). PHS78 - Persistence with glaucoma therapy in a lrage health organization in Israel. Value Health.

[B13] Levkovitch-Verbin H, Goldshtein I, Chodick G, Zigman N, Shalev V (2014). The Maccabi Glaucoma Study: prevalence and incidence of glaucoma in a large Israeli health maintenance organization. Am J Ophthalmol.

[B14] Pakravan M, Yazdani S, Javadi M-A, Amini H, Behroozi Z, Ziaei H, et al (2013). a population-based survey of the prevalence and types of glaucoma in Central Iran: The Yazd Eye Study. Ophthalmology.

[B15] Hashemi H, Mohammadi M, Zandvakil N, Khabazkhoob M, Hassan Emamiane M, Shariati M, et al (2018). Prevalence and risk factors of glaucoma in an adult population from Shahroud, Iran. J Curr Ophthalmol.

[B16] Al-Bahlal A, Khandekar R, Al Rubaie K, Alzahim T, Edward DP, Kozak I (2017). Changing epidemiology of neovascular glaucoma from 2002 to 2012 at King Khaled Eye Specialist Hospital, Saudi Arabia. Indian J Ophthalmol.

[B17] Al-Shaaln FF, Bakrman MA, Ibrahim AM, Aljoudi AS (2011). Prevalence and causes of visual impairment among Saudi adults attending primary health care centers in northern Saudi Arabia. Ann Saudi Med.

[B18] Khandekar R, Chauhan D, Yasir ZH, Al-Zobidi M, Judaibi R, Edward DP (2019). The prevalence and determinants of glaucoma among 40 years and older Saudi residents in the Riyadh Governorate (except the Capital) – a community based survey. Saudi J Ophthalmol.

[B19] Khandekar R, Mohammed AJ (2005). Visual disabilities among diabetics in Oman. Saudi Med J.

[B20] Kahloun R, Khairallah M, Resnikoff S, Cicinelli MV, Flaxman SR, Das A, et al

[B21] Cumoletti M https://www.migrationpolicy.org/article/middle-eastern-and-north-african-immigrants-united-states-2016.

[B22] Sit AJ (2014). Intraocular pressure variations: causes and clinical significance. Can J Ophthalmol.

[B23] Cho HK, Kee C (2014). Population-based glaucoma prevalence studies in Asians. Surv Ophthalmol.

[B24] Takamoto M, Araie M (2014). Genetics of primary open angle glaucoma. Jpn J Ophthalmol.

[B25] Nakano M, Ikeda Y, Taniguchi T, Yagi T, Fuwa M, Omi N, et al (2009). Three susceptible loci associated with primary open-angle glaucoma identified by genome-wide association study in a Japanese population. Proc Natl Acad Sci.

[B26] Kondkar AA, Azad TA, Almobarak FA, Abu-Amero KK, Al-Obeidan SA (2019). Polymorphism rs7961953 in TMTC2 gene is not associated with primary open-angle glaucoma in a Saudi cohort. Ophthalmic Genet.

[B27] Kondkar AA, Azad TA, Almobarak FA, Kalantan H, Sultan T, Al-Obeidan SA, et al (2017). Polymorphism rs11656696 in GAS7 is not associated with primary open angle glaucoma in a Saudi cohort. Genet Test Mol Biomark.

[B28] Kondkar AA, Sultan T, Almobarak FA, Kalantan H, Abu-Amero KK, Al-Obeidan SA (2018). Plexin domain containing 2 (PLXDC2) gene polymorphism rs7081455 may not influence POAG risk in a Saudi cohort. BMC Res Notes.

[B29] Kondkar AA, Edward NB, Kalantan H, Al-Kharashi AS, Altuwaijri S, Mohamed G, et al (2017). Lack of association between polymorphism rs540782 and primary open angle glaucoma in Saudi patients. J Negat Results BioMed.

[B30] Abu-Amero KK, Kondkar AA, Mousa A, Almobarak FA, Alawad A, Altuwaijri S, et al (2016). Analysis of cyclin-dependent kinase inhibitor-2B rs1063192 polymorphism in Saudi patients with primary open-angle glaucoma. Genet Test Mol Biomark.

[B31] Narooie-Nejad M, Rasouli A, Mousavi M, Rohani MR (2017). Study of MYOC gene mutation in POAG patients in Zahedan, Iran. Clin Lab.

[B32] Kondkar AA, Azad TA, Sultan T, Osman EA, Almobarak FA, Al-Obeidan SA

[B33] Kondkar AA, Azad TA, Almobarak FA, Kalantan H, Sultan T, Alsabaani NA, et al (2018). Polymorphism rs10483727 in the SIX1/SIX6 gene locus is a risk factor for primary open angle glaucoma in a Saudi cohort. Genet Test Mol Biomark.

[B34] Neamatzadeh H, Soleimanizad R, Atefi A, Zare-Shehneh M, Gharibi S, Shekari A, et al (2015). Association between p53 codon 72 (Arg72Pro) polymorphism and primary open-angle glaucoma in Iranian patients. Iran Biomed J.

[B35] Fakhraie G, Parvini F, Ghanavi J, Saif S, Farnia P (2020). Association of IL-10 gene promoter polymorphisms with susceptibility to pseudoexfoliation syndrome, pseudoexfoliative and primary open-angle glaucoma. BMC Med Genet.

[B36] Zhang YH, Xing YQ, Chen Z, Ma XC, Lu Q (2019). Association between interleukin-10 genetic polymorphisms and risk of primary open angle glaucoma in a Chinese Han population: a case-control study. Int J Ophthalmol.

[B37] The Genetics of Glaucoma in People of African Descent (GGLAD) Consortium (2019). Association of genetic variants with primary open-angle glaucoma among individuals with African ancestry. JAMA.

[B38] Tielsch JM, Katz J, Sommer A, Quigley HA, Javitt JC (1994). Family history and risk of primary open angle glaucoma. The Baltimore Eye Survey Arch Ophthalmol.

[B39] Yazdani S, Akbarian S, Pakravan M, Afrouzifar M (2015). Prevalence of angle closure in siblings of patients with primary angle-closure glaucoma. J Glaucoma.

[B40] Vajaranant TS, Nayak S, Wilensky JT, Joslin CE (2010). Gender and glaucoma: what we know and what we need to know. Curr Opin Ophthalmol.

[B41] Ryskulova A, Turczyn K, Makuc DM, Cotch MF, Klein RJ, Janiszewski R (2008). Self-reported age-related eye diseases and visual impairment in the United States: results of the 2002 national health interview survey. Am J Pub Health.

[B42] Shweikh Y, Ko F, Chan MPY, Patel PJ, Muthy Z, Khaw PT, et al (2015). Measures of socioeconomic status and self-reported glaucoma in the UK Biobank cohort. Eye.

[B43] Renard JP, Rouland JF, Bron A, Sellem E, Nordmann J-P, Baudouin C, et al (2013). Nutritional, lifestyle and environmental factors in ocular hypertension and primary open-angle glaucoma: an exploratory case-control study. Acta Ophthalmol.

[B44] Hashemi H, Kashi AH, Fotouhi A, Mohammad K (2005). Distribution of intraocular pressure in healthy Iranian individuals: the Tehran Eye Study. Br J Ophthalmol.

[B45] Gazzard G, Morgan W, Devereux J, Foster P, Oen F, Seah S, et al (2003). Optic disc hemorrhage in Asian glaucoma patients. J Glaucoma.

[B46] Beck RW, Messner DK, Musch DC, Martonyi CL, Lichter PR

[B47] Deb AK, Kaliaperumal S, Rao VA, Sengupta S (2014). Relationship between systemic hypertension, perfusion pressure and glaucoma: a comparative study in an adult Indian population. Indian J Ophthalmol.

[B48] Yusufali AM, Khatib R, Islam S, Alhabib KF, Bahonar A, Swidan HM, et al (2017). Prevalence, awareness, treatment and control of hypertension in four Middle East countries. J Hypertens.

[B49] Topouzis F, Coleman AL, Harris A, Jonescu-Cuypers C, Yu F, Mavroudis L, et al (2006). Association of blood pressure status with the optic disk structure in non-glaucoma subjects: the Thessaloniki Eye Study. Am J Ophthalmol.

[B50] Bowe A, Grunig M, Schubert J, Demir M, Hoffmann V, KÃ¼tting F, et al (2015). Circadian variation in arterial blood pressure and glaucomatous optic neuropathy–a systematic review and meta-analysis. Am J Hypertens.

[B51] Fan YY, Su WW, Liu CH, Chen HS-L, Wu S-C, Chang SHL, et al (2019). Correlation between structural progression in glaucoma and obstructive sleep apnea. Eye.

[B52] Wu X, Liu H (2015). Obstructive sleep apnea/hypopnea syndrome increases glaucoma risk: evidence from a meta-analysis. Int J Clin Exp Med.

[B53] Shi Y, Liu P, Guan J, Lu Y, Su K (2015). Association between glaucoma and obstructive sleep apnea syndrome: a meta-analysis and systematic review. PloS One.

[B54] Chaitanya A, Pai VH, Mohapatra AK, Ve RS (2016). Glaucoma and its association with obstructive sleep apnea: a narrative review. Oman J Ophthalmol.

[B55] Friedlander AH, Graves LL, Chang TI, Kawakami KK, Lee UK, Grabich SC, et al (2018). Prevalence of primary open-angle glaucoma among patients with obstructive sleep apnea. Oral Surg Oral Med Oral Pathol Oral Radiol.

[B56] Bagabas N, Ghazali W, Mukhtar M, AlQassas I, Merdad R, Maniyar A, et al (2019). Prevalence of glaucoma in patients with obstructive sleep apnea. J Epidemiol Glob Health.

[B57] Abuyassin B, Laher I (2016). Diabetes epidemic sweeping the Arab world. World J Diabetes.

[B58] Gerber AL, Harris A, Siesky B, Lee E, Schaab TJ, Huck A, et al (2015). Vascular dysfunction in diabetes and glaucoma: a complex relationship reviewed. J Glaucoma.

[B59] Al-Akily SA, Bamashmus MA, Gunaid AA (2011). Causes of visual impairment and blindness among Yemenis with diabetes: a hospital-based study. East Mediterr Health J.

[B60] Laville V, Kang JH, Cousins CC, Iglesias AI, Nagy R, Cooke Bailey JN (2019). Genetic correlations between diabetes and glaucoma: an analysis of continuous and dichotomous phenotypes. Am J Ophthalmol.

[B61] Global Tobacco Surveillance System Collaborating Group (2005). Global Tobacco Surveillance System (GTSS): purpose, production, and potential. J Sch Health.

[B62] Cherecheanu AP, Garhofer G, Schmidl D, Werkmeister R, Schmetterer L (2013). Ocular perfusion pressure and ocular blood flow in glaucoma. Curr Opin Pharmacol.

[B63] Choi J, Kook MS (2015). Systemic and ocular hemodynamic risk factors in glaucoma. Biomed Res Int.

[B64] Kim YJ, Kim JM, Shim SH, Bae JH, Park KH, The Epidemiologic Survey Committee of the Korean Ophthalmological S (2015). Associations between optic cup-to-disc ratio and systemic factors in the healthy Korean population. Korean J Ophthalmol.

[B65] Amerasinghe N, Wong TY, Wong WL, Mitchell P, Shen SY, Loon S-C, et al (2008). Determinants of the optic cup to disc ratio in an Asian population: the Singapore Malay Eye Study (SiMES). Arch Ophthalmol.

[B66] Kuang TM, Liu CJ, Ko YC, Lee SM, Cheng CY, Chou P (2014). Distribution and associated factors of optic disc diameter and cup-to-disc ratio in an elderly Chinese population. J Chin Med Assoc.

[B67] Mahboub B, Safarainni B, Alhariri H, Vats M (2013). Sleep breathing disorders in female population of Dubai, UAE. Health.

[B68] BaHammam AS, Alrajeh MS, Al-Jahdali HH, BinSaeed AA (2008). Prevalence of symptoms and risk of sleep apnea in middle-aged Saudi males in primary care. Saudi Med J.

[B69] Bahammam AS, Al-Rajeh MS, Al-Ibrahim FS, Arafah MA, Sharif MM (2009). Prevalence of symptoms and risk of sleep apnea in middle-aged Saudi women in primary care. Saudi Med J.

[B70] Khassawneh B, Ghazzawi M, Khader Y, Alomari M, Amarin Z, Shahrour B, et al (2008). Symptoms and risk of obstructive sleep apnea in primary care patients in Jordan. Sleep Breath.

[B71] Khazaie H, Maroufi A (2014). Obstructive sleep apnea syndrome; a neglected cause of traffic collision among Iranian public transport drivers. J Inj Violence Res.

[B72] Al-Nozha MM, Al-Maatouq MA, Al-Mazrou YY, Al-Harthi SS, Arafah MR, Khalil MZ, et al (2004). Diabetes mellitus in Saudi Arabia. Saudi Med J.

[B73] Al-Till MI, Al-Bdour MD, Ajlouni KM (2005). Prevalence of blindness and visual impairment among Jordanian diabetics. Eur J Ophthalmol.

[B74] Herman WH, Aubert RE, Engelgau MM, Thompson TJ, Ali MA, Sous ES, et al (2004). Diabetes mellitus in Egypt: glycaemic control and microvascular and neuropathic complications. Diabet Med.

[B75] Scharnweber G, TeachMideast, editor Teaching the Middle East: a resource guide for American educators. Teachmideast.org: Middle East Policy Council. p.

[B76] Pandova MG, Al-Merjan JI, Sadeq NA (2019). Registered blindness in Kuwait–15 years of dynamic changes. Ophthalmic Epidemiol.

